# Quercetin-3-O-β-D-glucopyranoside-rich fraction demonstrated efficacy against infectious, secretory, and osmotic models of diarrhoeal rats

**DOI:** 10.1186/s43141-023-00489-7

**Published:** 2023-03-21

**Authors:** Olalekan Bukunmi Ogunro, Emmanuel Bankole Ofeniforo, Aderonke Elizabeth Fakayode

**Affiliations:** 1 Department of Biological Sciences, KolaDaisi University, Ibadan, 200213 Nigeria; 2 Department of Biochemistry, University of Ilorin, Ilorin, 240222 Nigeria; 3 Department of Biochemistry, Federal University of Technology, Akure, Nigeria

**Keywords:** *Shigella flexneri*, Water-holding capacity, Acute gastroenteritis, Diarrhoea morbidity, Enterotoxin, Antimicrobial susceptivity

## Abstract

**Background:**

The prevalence of diarrhoea remains high despite efforts by governments and NGOs to reverse trend. This study investigated the antidiarrhoeal activity and mechanism of *Spondias mombin* leaf fraction rich in quercetin-3-O-β-D-glucopyranoside (Q3G-RF) because of the acclaimed therapeutic efficacy. Secretory, osmotic, and infectious diarrhoea models using castor oil, magnesium sulphate, and *Shigella flexneri* respectively were evaluated at the doses of 100, 200, and 400 mg/kg in Wistar rats. Enteropathy was induced with castor oil and magnesium sulphate, while gastrointestinal motility was determined with charcoal meal.

**Results:**

Findings showed no mortality after 14 days of experimental period and no significant changes in behaviour, food, and water consumption. Relative to control, Q3G-RF inhibited the three models of diarrhoea, enteropathy, and gastrointestinal motility; bacterial colonies were reduced by Q3G-RF, while it improved the relative body weight of the animals. Q3G-RF also increased the intestinal concentration/activity of glucose, total protein, and Na^+^–K^+^ ATPase but reduced the concentration of TNF-α, PGE_2_, IL-1β, nitric oxide, Na^+^, K^+^, and Cl^−^ in the diarrhoeal models. The intestinal fluid level of K^+^, Na^+^, and Cl^−^ was significantly decreased by Q3G-RF in the enteropathy model. Length of the small intestine in the motility model was also increased by Q3G-RF, while peristaltic index and inhibition of peristalsis were reduced.

**Conclusion:**

Overall, quercetin-3-O-β-D-glucopyranoside from *Spondias mombin* leaves demonstrated efficacy against infectious, secretory, and osmotic form of diarrhoeal and further justified its traditional use in the treatment of diarrhoea due to its antimotility, antisecretory, and antimicrobial properties by mechanism related to enhanced Na^+^–K^+^ ATPase, repressed nitric oxide, and suppressed prostaglandins.

## Background

Diarrhoea, with the characteristics of frequent passing of fluid faeces, increased gastrointestinal motility and secretion, decreased fluid absorption, and declination of electrolytes and water; is part of the main causes of death around the world. Relative to pneumonia, it is responsible for an estimated 40% of annual global child mortality [1]. About 5 million children under the age of 5 die every year from diarrhoea, i.e. one child every minute [1, 2]. Diarrhoea not only causes short-term health problems and premature death but also leads to malnutrition, which in turn lowers resistance to infection and stunts children’s physical development. Thus, diarrhoea is a major threat to public health because it can result in premature death, disability, and/or increased medical costs [3].

The prevalence of diarrhoea remains high despite efforts by governments and NGOs to reverse the trend. Fifteen countries account for about 75% of all diarrhoea-related deaths, and six of them (India, Nigeria, the Democratic Republic of the Congo, Afghanistan, Pakistan, and Angola) failed to meet MDG no. 4, which required a reduction of the mortality rate for children under 5 by two-thirds between 1990 and 2015. Ethiopia, Indonesia, Bangladesh, China, Niger, and Tanzania, all of which achieved their targets, remain in the top 15 due to the high prevalence of diarrhoea in their most vulnerable populations [4]. For this reason, a more efficient strategy for addressing diarrhoea is urgently needed in conformity with meeting the goal 3 of the Sustainable Development Agenda to end premature death in children younger than 5 years old by 2030.

Diarrhoea can be caused by a number of factors including alcohol, hormones or bile salts, intoxication, irritable bowel syndrome, secretory tumours, or intestinal microorganisms like *S. aureus*, *E. coli*, *C. albicans*, *S. typhi*, and *Shigella flexneri* [1]. Antibiotics and other synthetically produced drugs for treating diarrhoea often cause unpleasant side effects like toxicity, illusion, headache, convulsion, stomach spasm, disgorging, and constipation [5]. From evidence-based studies, the World Health Organization (WHO) endorses traditional medical practices for the treatment and prevention of diarrhoea because of their availability, economic viability, inherited expertise, and perceived efficacy [6]. Several plants and medicinal herbs have been entailed as remedy for diarrhoea in traditional knowledge of medicine in a few cultural backgrounds. It is therefore not a lost need to screen for antidiarrhoeal principles from medicinal plants like *Spondias mombin*. *Spondias mombin* Linn (Anacardiaceae) is tree having foliage that persists and remains green throughout the year and majorly found in tropical countries such as Nigeria, Cameroon, Mexico, Peru, Brazil, and Ivory Coast. In English, hog plum is the common name, while the Yoruba, Hausa, and Igbo tribes in Nigeria refer to it as *Akika etikan*/*Iyeye*, *Tsardarmasar*, and *Ichikara*, respectively [7]. Leaves, fruits, stem bark, seeds, pulp, roots, flowers, and even the entire plant itself have been used in traditional medicine for the prevention and treatment of a wide variety of conditions [8, 9]. Leaf and bark extract *S. mombin* contain a wide variety of chemical constituents not limited to sterols, tannins, flavonoids, quinones, saponins, or antioxidant chemicals [9, 10] ,while some pharmacological activity of the crude extract or active constituents has been reported both in vitro and in vivo [9, 11–21]. A detailed and comprehensive study on the nutritional, phytochemistry, bioactivity, and toxicity of *S. mombin* was reported by Ogunro et al. [22].

Although *S. mombin* leaves have been acclaimed to have antidiarrhoeal effects in some cultures, very little is known about it from the science point of view, including its likely mechanism of action. On the other hand, Ogunro et al. [23] reported the *S. mombin*’s quercetin-3-O-β-D-glucopyranoside as a promising agent of various therapeutic advantages. This informed the scope of this research on *Spondias mombin* leaf fraction rich in quercetin-3-O-β-D-glucopyranoside (Q3G-RF). This study therefore evaluated the antidiarrhoea efficacy and pharmacological mechanism of Q3G-RF from *S. mombin* leaves on infectious, osmotic, and secretory diarrhoea models using *Shigella flexneri*, magnesium sulphate, and castor oil, respectively, enteropathy model using magnesium sulphate and castor oil, and gastrointestinal motility model using charcoal meal. Biochemical indices like Na^+^–K^+^ ATPase, nitric acid, total protein, glucose, and sodium, potassium, and chloride ions in the intestinal fluid as well as its pH were used to evaluate the mode of action of Q3G-RF. Findings from the study will provide more scientific assertion on the antidiarrhoeal efficacy and the mechanism of action of Q3G-RF for its exploration as therapeutic agent in drug formulation against premature mortality, disability, and/or increased health-care costs associated with diarrhoea in children.

## Methods

### Plant sample and preparation

*Spondias mombin* leaves were harvested from a healthy plant in Isaba Ekiti, Ekiti State of Nigeria, after which it was authenticated by an expert botanist at the herbarium in the Department of Plant Biology of University of Ilorin. The voucher specimen was assigned with UILH/001/1147.

Samples of the leaves were dried on laboratory bench properly exposed to natural air until a constant weight was obtained. The dried leaves were grinded to smooth powder using a blender (Trident Ltd., China). Exactly 1000 g of the powdered sample was extracted in an airtight jar with 3 L of ethanol at 25 °C for 96 h during which the jar was regularly shaken. Extraction process with ethanol was done twice to get clear supernatant. Thereafter, fresh cotton bed was initially used to separate the obtained solution after which Whatman No.1 filters paper was used. After this, a rotary vacuum evaporator (operated at low pressure and temperature) was used to concentrate the obtained filtrate. The compact extract obtained was further fractionated using a modified partitioning guild of Kupchan [24]. Separated from the ethanol extract were the fractions of butanol, ethyl acetate, and hexane capable of being dissolved. The evaporated solvents yielded fractions of ethyl acetate (10.7), hexane (9.5), and butanol (6.9). First, ethyl acetate was extracted with silica gel, and then, the extract was fractionated with solvents of varying polarities using vacuum liquid chromatography (VLC). VLC (960 mg) with methanol (MeOH, 20%) in ethyl acetate washed with solvent mixtures of chloroform (CHCl_3_) and MeOH, and the resulting fraction (2.3 g) was analysed by gel filtration chromatography which produced 28 fractions. Thereafter, the subfractions from the fourth to sixteenth were added together to yield 450 mg. Q3G-RF was obtained through rinsing with methanol (20%) in mixture of ethyl acetate of the last step of column chromatography.

### Screening for phytochemical constituents

Leaf extract of *S. mombin* was analysed for alkaloid [25], anthraquinones [26], cardenolides and dienolides [27], cardiac glycoside [28], flavonoids and phenolics [29], phlobatannins [30], steroids [31], saponins [32], and tannins/terpenes [33]. For alkaloids, aqueous HCl (1% v/v) (5.0 cm^3^) and the leaf extract of *Spondias mombin* (1.0 cm^3^) were mixed on a water bath. Filtrate from the mixture was recovered when hot, while distilled water was added to the residue. A mixture constituted the filtrate (1.0 cm^3^), two drops each from potassium mercuric iodide solution (Mayer’s reagent), solution of iodine in potassium iodide (Wagner’s reagent), and solution of potassium bismuth iodide (Dragendorff’s reagent). A cream colour formed with potassium mercuric iodide solution and reddish-brown precipitate with solution of iodine in potassium iodide, and solution of potassium bismuth iodide confirmed the presence of alkaloids. To screen for anthraquinones, the leaf extract of *Spondias mombin* (3.0 cm^3^) was thoroughly mixed with benzene (10.0 cm^3^) and filtered. A known volume (5.0 cm^3^) of NH_4_OH (10% v/v) was then added to the resulting filtrate. The presence of anthraquinones was confirmed by a pink colour in the lower phase. Addition of leaf extract of *Spondias mombin* (5.0 cm^3^) to glacial acetic acid (2.0 cm^3^) having a drop of FeCl_3_ solution (5 %, w/v) after which concentrated H_2_SO_4_ (1.0 cm^3^) was added, while the presence of cardenolides was confirmed by ring, a characteristic of a deoxy sugar. For cardiac glycosides, *Spondias mombin* leaf extract (1.0 cm^3^) was mixed with chloroform (2.0 cm^3^), which was followed by the addition of H_2_SO_4_ (2.0 cm^3^). A part of aglycone of the cardiac glycosides was affirmed by a reddish-brown colour at the interface.

Exactly 1.0 cm^3^ of NaOH (10 % w/v) was mixed with the leaf extract of *Spondias mombin* (3.0 cm^3^). Flavonoids was confirmed by the formation of a tallow colour. Screening for phenolics, 5% w/v of FeCl_3_ (two drops), was mixed with the leaf extract of *Spondias mombin* leaf (1.0 cm^3^). The presence of phenolics was confirmed by a greenish precipitate.

Exactly 3.0 cm^3^ of the leaf extract of *Spondias mombin* with aqueous HCl (1%) and the presence of phlobatannins was ascertained red precipitate formed. For saponins, the leaf extract of *Spondias mombin* (5.0 cm^3^) was heated to boiling point in distilled water (20 cm^3^). A known volume of the obtained filtrate (10.0 cm^3^) was then added distilled water (5.0 cm^3^) and shaken vigorously. The presence of saponin was confirmed by the formation of stable persistent froth. Steroid presence in the leaf extract of *Spondias mombin* was confirmed by the red colour formed after five drops of concentrated H_2_SO_4_ were added to the extract (1.0 cm^3^). A white precipitate formed from the mixture of the leaf extract (1.0 cm^3^) was added to 1.0 cm^3^ of ethanolic KOH (10% w/v) confirmed the presence of tannins. A mixture formed by the addition of the leaf extract of *S. mombin* (1.0 cm^3^), 5 drops of acetic acid anhydride, and a drop of concentrated H_2_SO_4_ was steamed for an hour after which it was neutralized by addition of NaOH and then chloroform to form a bluish green colour that confirmed the presence of terpenes.

### Experimental rats and microorganism

Male Wistar rats used in the present study were of the average weight of 130.7 ± 5.14 g. They were initially made to adapt to a housing condition for the experiment for a 14-day period. The temperature, light period, and humidity were maintained at 39 °C, 12-h light/dark cycle, and 50% respectively in a proper aerated animal house. Rat pellets from Premier Feeds, Ibadan, and water from a running tap were provided ad libitum except when being fasted. Approval for the research (FNS/ERC/2022/020B) was granted by the Ajayi Crowther University’s Research Ethic Committee. In addition, handling and care for the rats were equally done in respect with the ethical rules in the “Care and Use of Laboratory Animals” compiled by National Academy of Science, National Institute of Health (Bethesda, MD, USA).

*Shigella flexneri*-related diarrhoeal infection was used for this study. The bacterial strain kept at 4 °C was sub-cultured prior to the tests.

### Acute toxicity examination

The Organisation for Economic Co-operation and Development (OECD)’s protocol for ascertaining the single-dose acute oral toxicity was used. In this study, five experimental groups that consisted of the control group and test groups comprised of six male Wistar rats randomly assigned into each group. Animals in the control group were treated with normal saline, while animals in the test groups were treated with 500 mg/kg body weight, 1000 mg/kg body weight, 2000 mg/kg body weight, and 5000 mg/kg body weight in single dose administered via oral route. Following the oral treatment, overall behaviour and signs of toxicity in the rats were observed repeatedly for 1 h and then intermittently at every 4 h over a period of 24 h. Furthermore, behavioural changes and signs of toxicity, food, and water intake as well as mortality rates were monitored daily up until 14 days for following treatment, whereas changes in body weight were noted on days 0, 7, and 14. The median lethal dose (*LD*_50_) was used to establish doses for the acute toxicity test [34].

### Procedure of experiment and treatment groups of the experimental rats

#### Diarrhoea study

##### Infectious diarrhoea study

For the infectious diarrhoeal study, *Shigella flexneri* was used to induce diarrhoea. A total of 70 male Wistar rats fasted overnight for 18 h (except for water) were arbitrarily allocated into 6 treatment groups for 14 days as follows:

- Group A: Normal saline (1 mL) — sham control
- Group B: Inoculum (2 mL) + normal saline (1 mL) — negative control
- Group C: Inoculum (2 mL) + ciprofloxacin (2.5 mgkg^−1^ body weight) — positive control
- Group D: Inoculum (2 mL) + Q3G-RF (100 mgkg^−1^ body weight)
- Group E: Inoculum (2 mL) + Q3G-RF (200 mgkg^−1^ body weight)
- Group F: Inoculum (2 mL) + Q3G-RF (400 mgkg^−1^ body weight)

Rat chows were introduced ad libitum from the 4–6th h. McFarland 3 standard inoculum (at 9 × 10^8^ CFU/mL) was administered orally prior to oral treatment with either normal saline, ciprofloxacin, or Q3G-RF following the confirmation of diarrhoeal [35]. Bacterial load was evaluated in the stools of inoculum-treated animals collected in sterile containers. Dissolution of 0.5-g faeces used in the study was done using 0.9% NaCl solution (5 mL) after which a portion (250 µl) of the obtained suspension mixture was added to 9750 mL of NaCl to obtain a diluted concentration. Culturing of each sample of the stool was done on a *Shigella-Salmonella* agar using 50 µl of the resultant suspension. After incubation for 18 h at 37 °C, the bacterial load was calculated and expressed as the number of colony-forming units (CFU) per gram of faeces per animal. Relative body weight was also monitored during the treatment period.

##### Secretory diarrhoea study

For secretory diarrhoea study, diarrhoea was induced using castor oil. The animals totaling 70 male Wistar rats were fasted overnight for 18 h (asides water) and subjectively allocated to 6 treatment groups as follows:

- Group A: Normal saline (1 mL) — sham control
- Group B: Castor oil (1 mL) + normal saline (1 mL) — negative control
- Group C: Castor oil (1 mL) + loperamide (3 mgkg^−1^ body weight) — positive control
- Group D: Castor oil (1 mL) + Q3G-RF (100 mgkg^−1^ body weight)
- Group E: Castor oil (1 mL) + Q3G-RF (200 mgkg^−1^ body weight)
- Group F: Castor oil (1 mL) + Q3G-RF (400 mgkg^−1^ body weight)

##### Osmotic diarrhoea study

For osmotic diarrhoea study, magnesium sulphate was used to induce diarrhoea in the animals. A total of 70 male Wistar rats that were fasted over the night for 18 h (safe for water) were assigned to 6 treatment groups in a random manner as follows:

- Group A: Normal saline (1 mL) — sham control
- Group B: Magnesium sulphate (1 mL) + normal saline (1 mL) — negative control
- Group C: Magnesium sulphate (1 mL) + loperamide (3 mgkg^−1^ body weight) — positive control
- Group D: Magnesium sulphate (1 mL) + Q3G-RF (100 mgkg^−1^ body weight)
- Group E: Magnesium sulphate (1 mL) + Q3G-RF (200 mgkg^−1^ body weight)
- Group F: Magnesium sulphate (1 mL) + Q3G-RF (400 mgkg^−1^ body weight)

Either castor oil or magnesium sulphate was administered orally 1 h prior to oral treatment with either normal saline, loperamide, or Q3G-RF following the confirmation of diarrhoea. Assessment was done for every hour of six to determine the severity of diarrhoea based on the time diarrhoea begins, inhibition of diarrhoeal defecation in percentage, total faeces recorded, and the weight of the diarrhoeal faeces. The difference obtained from the fresh stool weight and an already weighed Whatman filter paper was recorded. Faecal weight was determined from a constant faecal weight obtained by oven-drying the new stool at 100 °C. The difference between the wet and dry weights of the faeces was used to determine the amount of water in the faeces [36]. Animals were sacrificed 6 h after treatment to collect small intestine for supernatants.

For the infectious, secretory, and osmotic model of diarrhoea, the experimental rats were housed in metabolic cages lined with uncontaminated preweighed Whatman filter (replaced every hour).

#### Enteropooling study

Castor oil model of enteropooling in the present study involved a total of 70 male rats fasted overnight for 18 h (except water intake) that were allotted into 6 groups randomly. They were administered with the required regimen as follows:

- Group A: Normal saline (1 mL) — sham control
- Group B: Castor oil (1 mL) + normal saline (1 mL) — negative control
- Group C: Castor oil (1 mL) + loperamide (3 mgkg^−1^ body weight) — positive control
- Group D: Castor oil (1 mL) + Q3G-RF (100 mgkg^−1^ body weight)
- Group E: Castor oil (1 mL) + Q3G-RF (200 mgkg^−1^ body weight)
- Group F: Castor oil (1 mL) + Q3G-RF (400 mgkg^−1^ body weight)

For the magnesium sulphate model of enteropooling, 70 rats that were fasted over the night for 19 h (asides water) were arbitrarily grouped into 6. They were treated with the different regimens as follows:

- Group A: Normal saline (1 mL) — sham control
- Group B: Magnesium sulphate (1 mL) + normal saline (1 mL) — negative control
- Group C: Magnesium sulphate (1 mL) + atropine sulphate (3 mgkg^−1^ body weight) — positive control
- Group D: Magnesium sulphate (1 mL) + Q3G-RF (100 mgkg^−1^ body weight)
- Group E: Magnesium sulphate (1 mL) + Q3G-RF (200 mgkg^−1^ body weight)
- Group F: Magnesium sulphate (1 mL) + Q3G-RF (400 mgkg^−1^ body weight)

Either castor oil or magnesium sulfate was administered orally 1 h prior to oral treatment with either normal saline, loperamide, or Q3G-RF. Treatment lasted for 2 h after which the rats were sacrificed. The weighed small intestine end at the pylorus and caecum was bound securely with a twin after which it was dissected to obtain its content into a graduated cap. Computational analysis of the volume and the masses obtained was used to derive the percentage suppression of the intestinal content. The small intestine was again weighed to compute the difference in the weight of a full and empty intestines [37].

### Gastrointestinal motility using charcoal

The animals totaling 70 male Wistar rats fasted overnight for a period of 18 h (but only water) were allocated into 6 groups randomly. They were treated with the specified regimen as follows:

- Group A: Normal saline (1 mL) — sham control
- Group B: Castor oil (1 mL) + normal saline (1 mL) + charcoal meal (1 mL) — negative control
- Group C: Castor oil (1 mL) + atropine sulphate (5 mgkg^−1^ body weight) + charcoal meal (1 mL) — positive control
- Group D: Castor oil (1 mL) + Q3G-RF (100 mgkg^−1^ body weight) + charcoal meal (1 mL)
- Group E: Castor oil (1 mL) + Q3G-RF (200 mgkg^−1^ body weight) + charcoal meal (1 mL)
- Group F: Castor oil (1 mL) + Q3G-RF (400 mgkg^−1^ body weight) + charcoal meal (1 mL)

Castor oil was administered orally 1 h prior to either normal saline (orally), atropine sulphate (intramuscularly), and Q3G-RF (orally). Thirty minutes afterwards, charcoal meal made from charcoal suspension in agarose agar was orally administered [38]. After 45 min of charcoal meal, all the rats were then sacrificed by dislocating the cervical vertebra [23]. The distance travelled by the charcoal meal through the intestine (from the pylorus to caecum) was measured and expressed as a percentage of gastrointestinal motility (distance moved) by the charcoal meal in ratio to the length of the small intestine (cm). Percentage inhibition of peristalsis produced by Q3G-RF was also calculated.

### Assay of selected biochemical parameters

From each group, 6 rats were sacrificed by dislocating the cervical vertebra [23] while carefully excising the small intestine to avoid being stretched. The technique highlighted by Yakubu and Salimon [38] was followed to prepare supernatants from the small intestine. Potassium, chloride, and sodium ions as well as total protein, nitric oxide, glucose, and Na^+^–K^+^ ATPase were assayed according to procedural guidelines by Tietz [39], Gornal et al*.* [40], Wo et al. [41], Barham and Trinder [42], and Bewaji et al. [43]. Tissue levels of TNF-α, PGE_2_, and IL-1β were estimated using commercially available ELISA kits from ABCAM Scientific Corporation (UK) and performed as per the manufacturer’s instructions.

### pH of intestinal fluid secretion

In the present study, pH cooperative strips that were ranged from 5 to 9 were used to read the intestinal fluid’s pH. The pH was measured by comparing the colour of the pH paper after dipping it into the sample with a standard [44].

### Statistical data analysis

Data were presented as the mean of six independent measurements ± SEM analysed with the Duncan’s multiple range test. Test values were compared to the control group using one-way and two analysis of variance (ANOVA) and then by Dunnett’s post hoc test, while statistically significant differences were set at *p* < 0.05 threshold (at the 95% confidence level). This study’s statistical analysis was performed using SPSS 20.0. (SPSS Inc., Chicago, IL, USA) and using the GraphPad Prism 5.0 software.

## Results

### Phytochemical constituents of *Spondias mombin* leaves

*Spondias mombin* leaves contained alkaloids, anthraquinones, flavonoids, phenolic, saponins, steroids, tannins, and triterpenes (Table 1).

**Table 1:** Secondary metabolite constituents of *Spondias mombin* leaf

**Secondary metabolites**	**Observation**	**Concentration (mg/ml)**
Alkaloids	Cream colour with Mayer’s reagent; reddish-brown with Wagner’s reagent	3.40 ± 0.10
Anthraquinones	Absence of bright pink colour with NH_3_	Not detected
Cardenolides and dienolides	Absence of turbid brown ring at the interface	Not detected
Cardiac glycosides	Brown-violet ring at the interface	0.01 ± 0.00
Chalcone	Absence of red coloration with concentrated sulphuric acid	Not detected
Phlobatannins	Absence of reddish precipitate with HCl	Not detected
Saponins	Stable, persistent froth with distilled water	4.80 ± 0.35
Steroids	Presence of violet to blue or green colour with H_2_SO_4_	0.92 ± 0.09
Tannins	Greenish-brown precipitate	1.47 ± 0.06
Terpenoids	Absence of reddish-brown colour with H_2_SO_4_	Not detected
Total flavonoids	Dark yellow precipitate with NH_3_	2.80 ± 0.36
Total phenolics	Greenish precipitate with FeCl_3_	1.73 ± 0.19

Values are means of three determinations ± SEM

### Acute toxicity study

Table 2 depicts the effects of test fraction, Q3G-RF from the leaves of the *Spondias mombin* in rats after acute oral administration. Q3G-RF at 500, 1000, and 2000 mg/kg body weight did not produce significant changes in behaviour, breathing, defecation, skin colour or hair loss, and postural abnormalities. Furthermore, there was no traces of toxicity, such as prolonged disorder of eating due to loss of appetite or hypoactivity. However, rats treated with 5000 mg/kg body weight of Q3G-RF exhibited sluggish movement and impaired breathing as well as anorexia and hypoactivity which lasted 15 min following drug treatment, but no mortality was recorded.

**Table 2:** Mortality and clinical signs of acute toxicity of Q3G-RF from *S. mombin* leaves administered orally to rats

**Route of administration**	**Dose (mg/kg body weight)**	**Mortality**		**Toxicity symptoms**
		**D/T**	**Latency (hour)**	
Oral	0	0/6	–	None
Oral	500	1/6	< 2 h	None
Oral	1000	2/6	< 2 h	None
Oral	2000	3/6	< 2 h	None
Oral	5000	4/6	< 1 h	Hyperactivity and prolonged disorder of eating due to loss of appetite

#### Effects of Q3G-RF from S. mombin leaves in S. flexneri-induced diarrhoeal rats

**Fig. 1:**
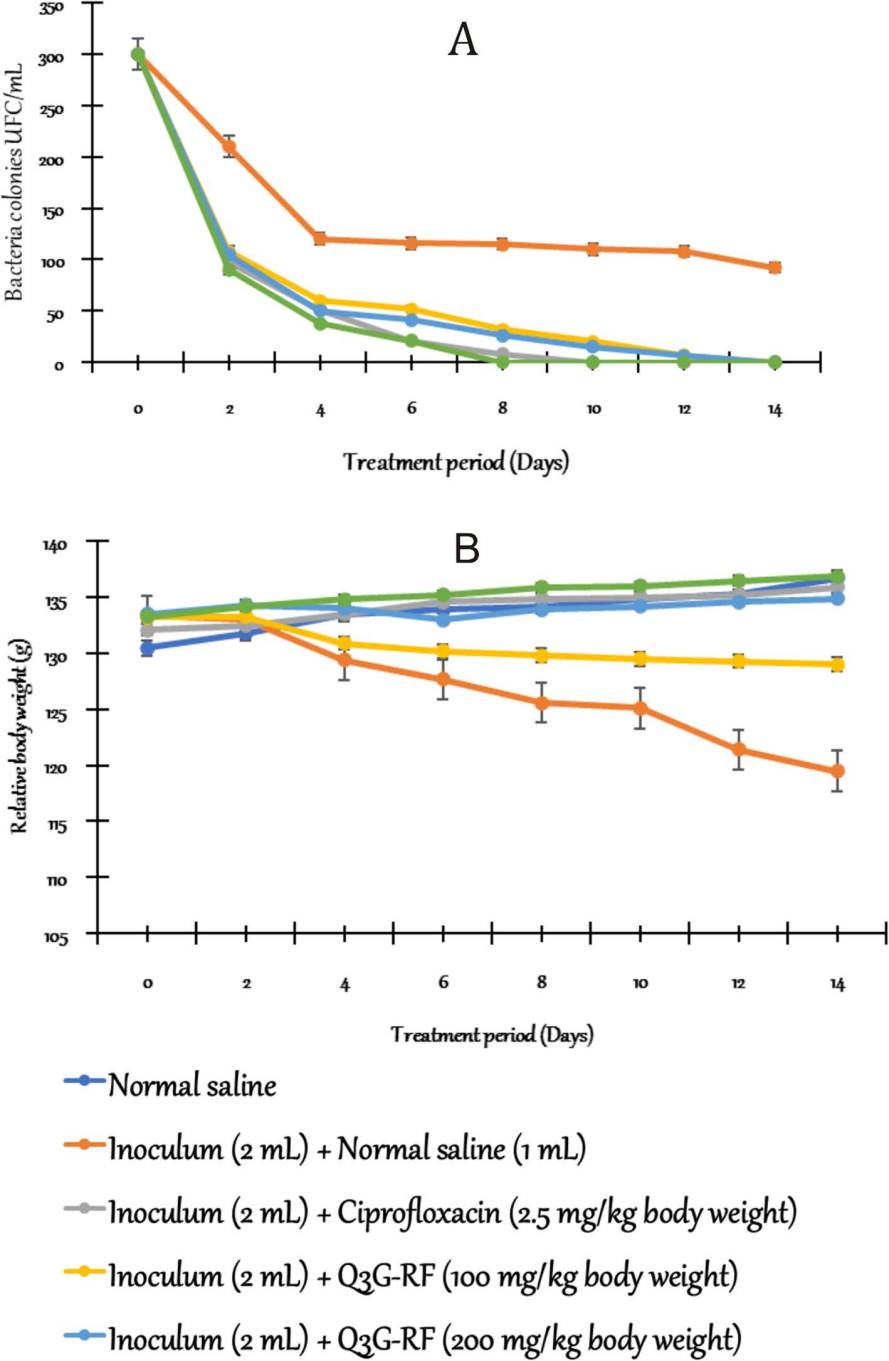
In vivo antibacterial activity of Q3G-RF from *S. mombin* leaves on *S. flexneri*-induced diarrhoea over 14 days of treatment. Number of *Shigella flexneri* in stool (**A**) and variation of the relative body weight (**B**) of male Wistar rats treated with normal saline, inoculum (2 mL) + normal saline (1 mL), inoculum (2 mL) + ciprofloxacin (2.5 mg/kg body weight), inoculum (2 mL) + Q3G-RF (100 mg/kg body weight), inoculum (2 mL) + Q3G-RF (200 mg/kg body weight), and inoculum (2 mL) + Q3G-RF (400 mg/kg body weight). Values are expressed as mean ± standard error of mean (*n* = 6). Significant differences from the sham control group are indicated by † and from negative control by #, *p* < 0.05

**Fig. 2:**
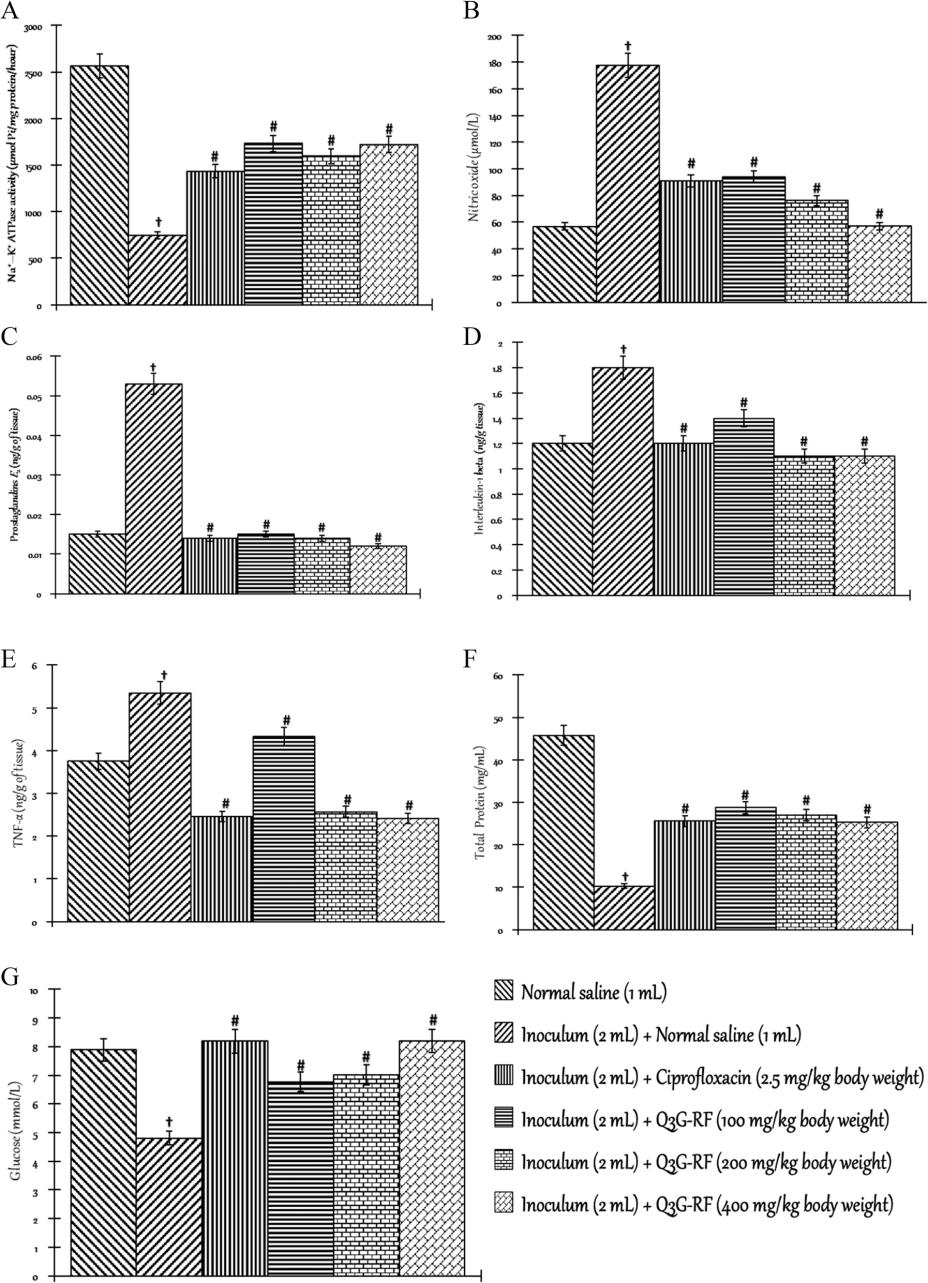
Effects of Q3G-RF from *S. mombin* leaves on selected biochemical parameters in *S. flexneri*-induced diarrhoeal rats after 14 days of treatment. Na^+^–K^+^ ATPase activity (**A**), nitric oxide level (**B**), prostaglandins *E*_2_ level (**C**), interleukin-1 beta level (**D**), TNF-α level (**E**), total protein level (**F**), and glucose level (**G**) in the intestinal tissue of male Wistar rats treated with normal saline, inoculum (2 mL) + normal saline (1 mL), inoculum (2 mL) + ciprofloxacin (2.5 mg/kg body weight), inoculum (2 mL) + Q3G-RF (100 mg/kg body weight), inoculum (2 mL) + Q3G-RF (200 mg/kg body weight), and inoculum (2 mL) + Q3G-RF (400 mg/kg body weight). Values are expressed as mean ± standard error of mean (*n* = 6). Significant differences from the sham control group are indicated by † and from negative control by #, *p* < 0.05

**Fig. 3:**
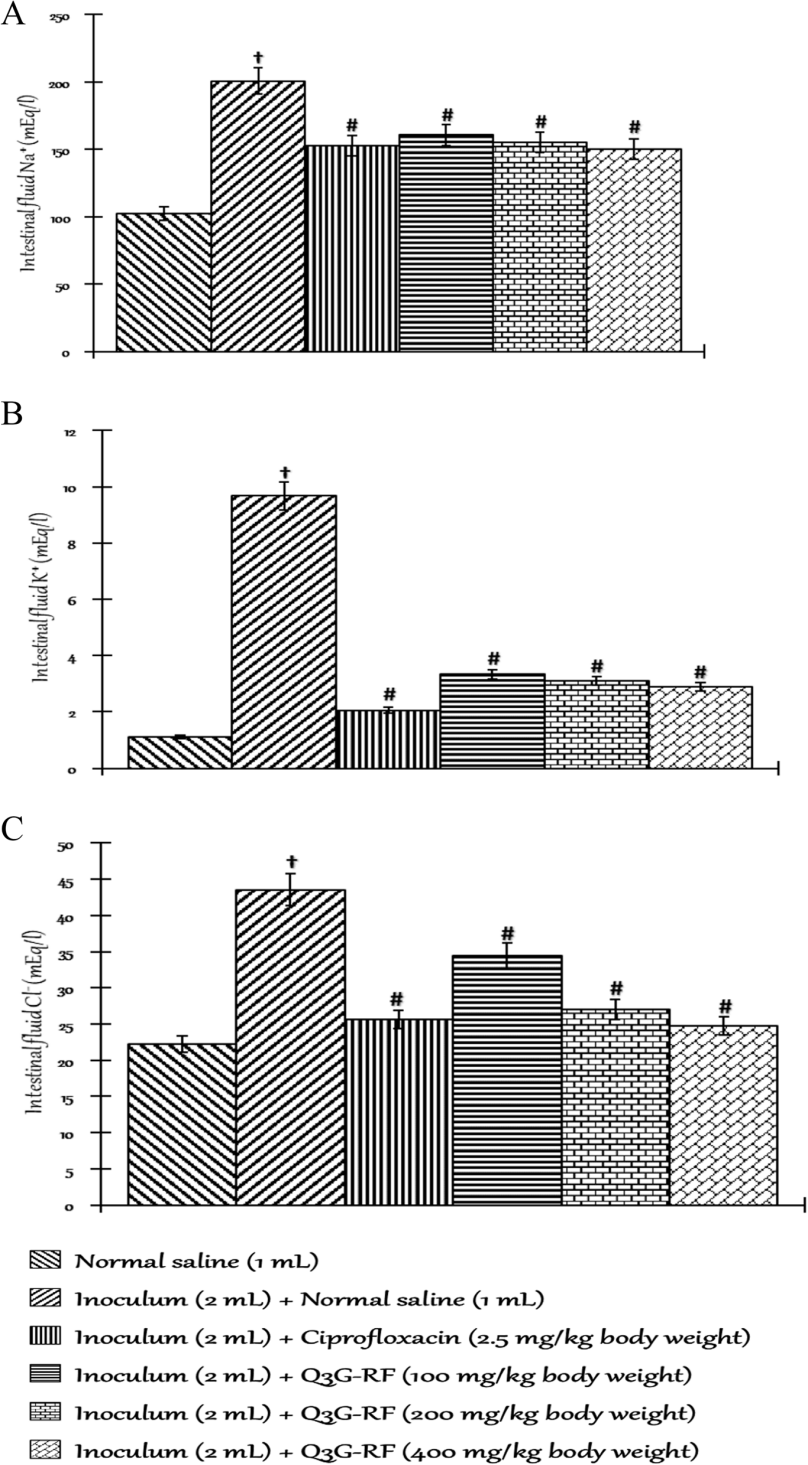
Effects of Q3G-RF from *S. mombin* leaves on electrolytes in *S. flexneri*-induced diarrhoeal rats after 14 days of treatment. Sodium ion concentration (**A**), potassium ion concentration (**B**), chloride ion concentration (**C**) in the intestinal fluid of male Wistar rats treated with normal saline, inoculum (2 mL) + normal saline (1 mL), inoculum (2 mL) + ciprofloxacin (2.5 mg/kg body weight), inoculum (2 mL) + Q3G-RF (100 mg/kg body weight), inoculum (2 mL) + Q3G-RF (200 mg/kg body weight), and inoculum (2 mL) + Q3G-RF (400 mg/kg body weight). Values are expressed as mean ± standard error of mean (*n* = 6). Significant differences from the sham control group are indicated by † and from negative control by #, *p* < 0.05

The effects of Q3G-RF from *S. mombin* leaves in rats induced into diarrhoeal by *S. flexneri* after 14 days is depicted in Figs. 1, 2, and 3.

There was significant increase (*p* < 0.05) in the number of bacterial colonies in the faeces of animals treated with *S. flexneri* inoculum and normal saline relative to the sham control treated with only normal saline (Fig. 1A). However, co-exposure to *S. flexneri* inoculum and Q3G-RF (100, 200, and 400 mg/kg body weight) or inoculum and ciprofloxacin (2.5 mg/kg body weight) significantly (*p* < 0.05) decreased the number of bacterial colonies in faeces of the animals (Fig. 1A). Q3G-RF at 400 mg/kg body weight demonstrated the maximum decrease of bacterial colonies in animals where the number of colonies was decreased from 90.12 ± 3.11 × 10^2^ UFC/g faeces at day 2 to 0.00 ± 0.00 × 10^2^ UFC/g faeces (100%) at day 8 of treatment compared with diarrhoeal animals treated with ciprofloxacin, whereas the number of colonies of 98.83 ± 5.78 × 10^2^ UFC/g faeces on day 2 decreased to 0.00 ± 0.00 × 10^2^ UFC/g faeces (100%) on day 10 of treatment and, relative to the bacteria colonies, decreased from 210.8 ± 51.37 × 10^2^ UFC/g faeces on the day 2 to 92.43 ± 6.20 × 10^2^ UFC/g faeces on day 14 in diarrhoeal animals treated with normal saline. Furthermore, there was no bacterial colonies in non-diarrhoeal animals throughout the treatment period (Fig. 1A).

The body weight of infected animals treated with normal saline was reduced significantly compared to the body weights of noninfected animals that received just normal saline (Fig. 1B, p < 0.05). In contraction, the body weight of animals co-exposed to *S. flexneri* inoculum and Q3G-RF (100, 200 and 400 mg/kg body weight) was significantly increased when compared to infected animals treated with normal saline (Fig. 1B). In the same manner, co-exposure to *S. flexneri* inoculum and ciprofloxacin was significantly increased (*p* < 0.05) when compared to infected animals treated with normal saline (Fig. 1B).

Relative to the sham control administered with normal saline only, *S. flexneri* significantly (*p* < 0.05) decreased the activity/concentration of Na^+^–K^+^ ATPase (Fig. 2A), total protein (Fig. 2F), and glucose (Fig. 2G) evaluated in the intestinal tissue but increased the intestinal tissue concentrations of nitric oxide (Fig. 2B), prostaglandins *E*2 level (Fig. 2C), interleukin-1 beta (Fig. 2D), and TNF-α (Fig. 2E). However, in animals co-exposed to *S. flexneri* inoculum and Q3G-RF (100, 200, and 400 mg/kg body weight) or inoculum and ciprofloxacin (2.5 mg/kg body weight), there was significant (*p* < 0.05) increase in the activity/concentration of Na^+^–K^+^ ATPase (Fig. 2A), total protein (Fig. 2F), and glucose (Fig. 2G) evaluated in the intestinal tissue but decreased the intestinal tissue concentrations of nitric oxide (Fig. 2B), prostaglandins *E*2 level (Fig. 2C), interleukin-1 beta (Fig. 2D), and TNF-α (Fig. 2E) when compared to the non-diarrhoeal animals treated with only normal saline (*p* < 0.05).

Furthermore, the intestinal fluid concentrations of Na^+^ (Fig. 3A), K^+^ (Fig. 3A), and Cl^−^ (Fig. 3A) were significantly increased by *S. flexneri* when compared to the non-diarrhoeal animals treated with only normal saline. Contrastingly, the intestinal fluid concentrations of Na^+^ (Fig. 3A), K^+^ (Fig. 3A), and Cl^−^ (Fig. 3A) were significantly (*p* < 0.05) decreased in animals co-exposed to *S. flexneri* inoculum and Q3G-RF (100, 200, and 400 mg/kg body weight) or inoculum and ciprofloxacin (2.5 mgkg^−1^ body weight), compared to the non-diarrhoeal animals administered normal saline only.

### Effects of Q3G-RF from S. mombin leaves in castor oil-induced diarrhoeal rats

**Fig. 4:**
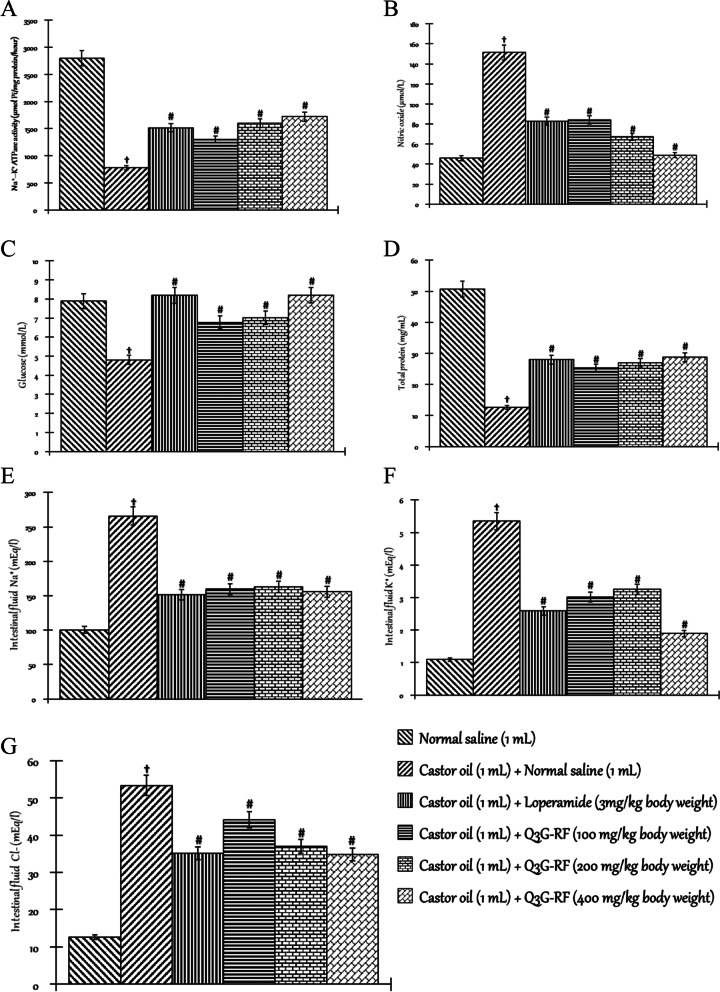
Effects of Q3G-RF from *S. mombin* leaves in castor oil-induced diarrhoeal rats after 6 h of treatment. Na^+^–K^+^ ATPase activity (**A**), nitric oxide level (**B**), total protein level (**C**), glucose level (**D**), sodium ion concentration (**E**), potassium ion concentration (**F**), chloride ion concentration (**G**) in the intestinal tissue/fluid of male Wistar rats treated with normal saline, castor oil (1 mL) + normal saline (1 mL), castor oil (1 mL) + loperamide (3 mg/kg body weight), castor oil (1 mL) + Q3G-RF (100 mg/kg body weight), castor oil (1 mL) + Q3G-RF (200 mg/kg body weight), and castor oil (1 mL) + Q3G-RF (400 mg/kg body weight). Values are expressed as mean ± standard error of mean (*n* = 6). Significant differences from the sham control group are indicated by † and from negative control by #, *p* < 0.05

The effects of Q3G-RF from *S. mombin* leaves in rats induced into diarrhoeal by castor oil are presented in Table 3 and Fig. 4. Relative to the sham control treated with only normal saline, castor oil shortened the onset time of diarrhoea with concomitant increase in the number of wet faeces, total faecal number, faecal fresh weight, and water content (*p* < 0.05). However, in animals co-exposed to castor oil and Q3G-RF (100, 200, and 400 mg/kg body weight) or castor oil and loperamide (3 mg/kg body weight), onset time of diarrhoea was prolonged, while the number of wet faeces, total faecal number, faecal fresh weight, and water content was significantly (*p* < 0.05) decreased when compared to the diarrhoeal animals treated with only normal saline (Table 3). Also, defecation inhibition in diarrhoeal animals treated with only normal saline was 0% when compared with the animals treated with only normal saline. However, co-exposure to castor oil and Q3G-RF at 100, 200, and 400 mg/kg body weight, respectively, inhibited defecation by value of 67%, 80%, and 86%, while co-exposure to castor oil and loperamide hydrochloride (3 mg/kg body weight) inhibited defecation by value of 85% when compared to the diarrhoeal animals treated with only normal saline (Table 3). Furthermore, castor oil significantly (*p* < 0.05) decreased the activity/concentration of Na^+^–K^+^ ATPase (Fig. 4A), total protein (Fig. 4C), and glucose (Fig. 4D) evaluated in the intestinal tissue but increased the intestinal tissue concentrations of nitric oxide (Fig. 4B), Na^+^ (Fig. 4E), K^+^ (Fig. 4F), and Cl^−^ (Fig. 4G), when compared to the non-diarrhoeal animals treated with only normal saline (*p* < 0.05). However, the intestinal activity/concentration of Na^+^–K^+^ ATPase (Fig. 4A), total protein (Fig. 4C), and glucose (Fig. 4D) were significantly (*p* < 0.05) increased, while the concentrations of nitric oxide (Fig. 4B), Na^+^ (Fig. 4E), K^+^ (Fig. 4F), and Cl^−^ (Fig. 4G) in the intestinal fluid were significantly (*p* < 0.05) reduced in animals co-exposed to castor oil and Q3G-RF (100, 200, and 400 mg/kg body weight) or castor oil and loperamide (3 mg/kg body weight). The 400 mg/kg body weight of Q3G-RF co-exposed to castor oil was more profound relative to other treatment groups and the loperamide group (Fig. 4).

**Table 3:** Some parameters of diarrhoea modulated by Q3G-RF from *S. mombin* leaves in castor oil model of Wistar rats

	**Non-diarrhoeal**	**Diarrhoel rats (1 mL of castor oil)**				
	**Normal saline**	**Normal saline**	**Loperamide**	**Q3G-RF from ** ***S. mombin*** ** leaves (mg/kg body weight)**		
	**1 mL**	**1 mL**	**3 mg/kg body weight**	**100**	**200**	**400**
Onset time (minutes)	54.45 ± 1.01	58.15 ± 1.05^b^	121.78 ± 1.80#	75.68 ± 1.17#	92.19 ± 1.19#	128.77 ± 1.82#
Total number of faeces	1.10 ± 0.11	5.80 ± 0.15^b^	2.00± 0.12#	3.90 ± 0.17#	2.80 ± 0.16#	2.00 ± 1.10#
Number of wet faeces	0.10 ± 0.03	5.10 ± 0.13^b^	0.70 ± 0.03#	1.60 ± 0.02#	0.90 ± 0.04#	0.70 ± 0.02#
Fresh weight of faeces (g)	0.19 ± 0.10	3.59 ± 0.11^b^	0.51 ± 0.07#	2.11 ± 0.10#	0.90 ± 0.05#	0.58 ± 0.05#
Water content of faeces (mL)	0.02 ± 0.00	1.88 ± 0.01^b^	0.41 ± 0.04#	1.01 ± 0.08#	0.67 ± 0.11#	0.45 ± 0.03#
Inhibition of defecation (%)	-	0	85	67	80	86

Values are mean of 6 replicates ± SEM, significant differences from the sham control group are indicated by ^b^ and from negative control by #, *p* < 0.05

### Effect of Q3G-RF from S. mombin leaves in magnesium sulphate-induced diarrhoeal rats

**Fig. 5:**
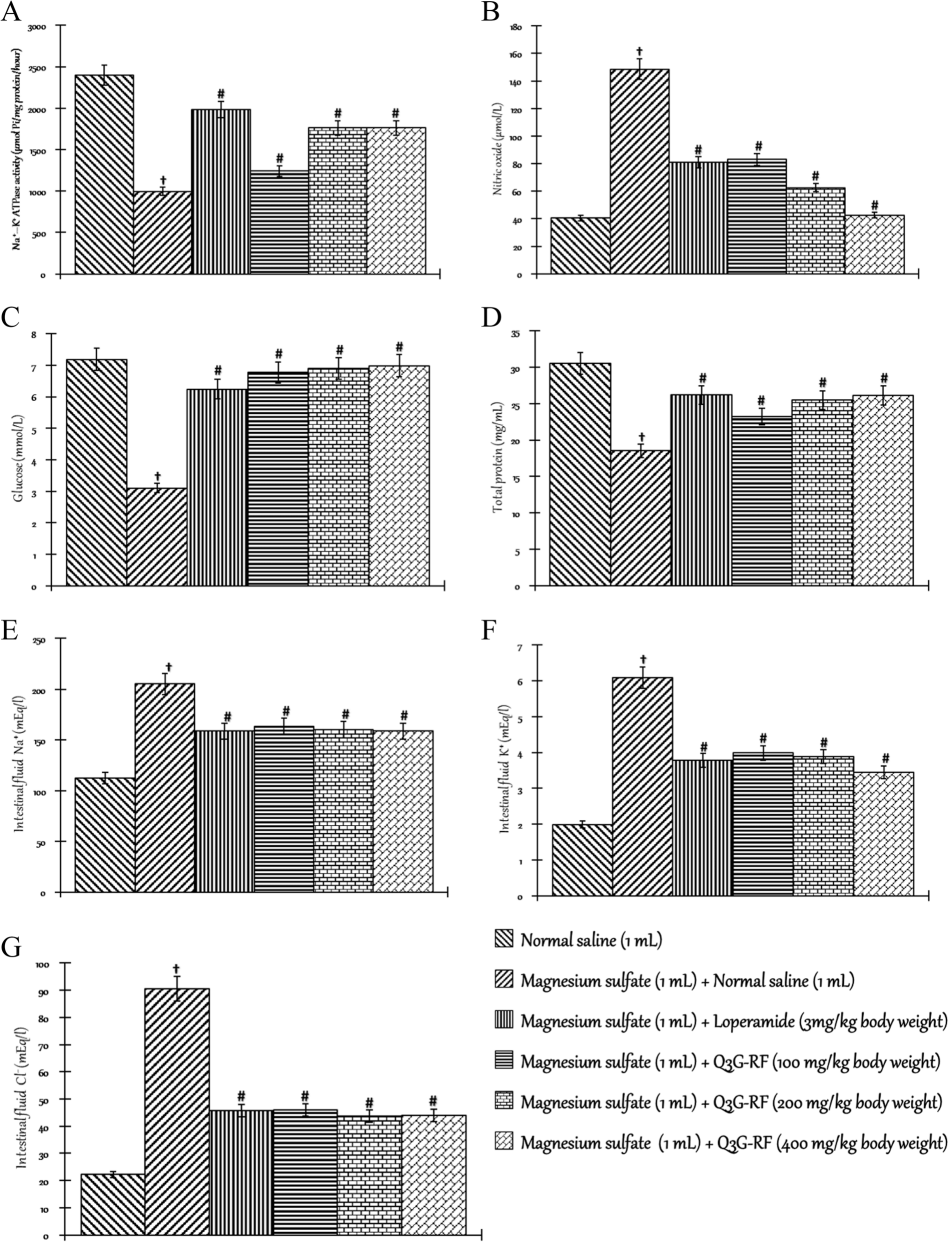
Effects of Q3G-RF from *S. mombin* leaves in magnesium sulphate-induced diarrhoeal rats after 6 h of treatment. Na^+^–K^+^ ATPase activity (**A**), nitric oxide level (**B**), total protein level (**C**), glucose level (**D**), sodium ion concentration (**E**), potassium ion concentration (**F**), chloride ion concentration (**G**) in the intestinal tissue/fluid of male Wistar rats treated with normal saline, magnesium sulphate (1 mL) + normal saline (1 mL), magnesium sulphate (1 mL) + loperamide (3 mg/kg body weight), magnesium sulphate (1 mL) + Q3G-RF (100 mg/kg body weight), magnesium sulphate (1 mL) + Q3G-RF (200 mg/kg body weight), and magnesium sulphate (1 mL) + Q3G-RF (400 mg/kg body weight). Values are expressed as mean ± standard error of mean (*n* = 6). Significant differences from the sham control group are indicated by † and from negative control by #, *p* < 0.05

Table 4 and Fig. 5 show the effect of Q3G-RF from *S. mombin* leaves on rats induced into diarrhoeal with magnesium sulphate. Relative to the non-diarrhoeal animals treated with only normal saline, magnesium sulphate shortened the onset time of diarrhoea with concomitant increase in the number of wet faeces, total faecal number, faecal fresh weight, and water content (*p* < 0.05). However, in animals co-exposed to magnesium sulphate and Q3G-RF (100, 200, and 400 mg/kg body weight) or magnesium sulphate and loperamide (3 mg/kg body weight), onset time of diarrhoea was prolonged, while the number of wet faeces, total faecal number, faecal fresh weight, and water content (*p* < 0.05) decreased when compared to the diarrhoeal animals treated with only normal saline (Table 4). Also, defecation inhibition in diarrhoeal animals treated with only normal saline was 0% when compared with the non-diarrhoeal animals treated with only normal saline. However, co-exposure to magnesium sulphate and Q3G-RF at 100, 200, and 400 mgkg^−1^ body weight, respectively, inhibited diarrhoeal faeces by value of 64%, 86%, and 100%, while co-exposure to magnesium sulphate and loperamide hydrochloride (3 mg/kg body weight) inhibited defecation by value of 85% when compared to the diarrhoeal animals treated with only normal saline (Table 4). In addition, intestinal activity/concentration of Na^+^–K^+^ ATPase (Fig. 5A), total protein (Fig. 5C), and glucose (Fig. 5D) was significantly decreased, while intestinal fluid concentrations of nitric oxide (Fig. 5B), Na^+^ (Fig. 5E), K^+^ (Fig. 5F), and Cl^−^ (Fig. 5G) were increased in the diarrhoeal animals treated with normal saline when compared to the non-diarrhoeal animals treated with only normal saline (*p* < 0.05). However, the intestinal activity/concentration of Na^+^–K^+^ ATPase (Fig. 5A), total protein (Fig. 5C), and glucose (Fig. 5D) was significantly (*p* < 0.05) increased, while the concentrations of nitric oxide (Fig. 5B), Na^+^ (Fig. 5E), K^+^ (Fig. 5F), and Cl^−^ (Fig. 5G) in the intestinal fluid were significantly (*p* < 0.05) reduced in animals co-exposed to magnesium sulphate and Q3G-RF (100, 200, and 400 mg/kg body weight) or magnesium sulphate and loperamide (3 mg/kg body weight). The efficacy of Q3G-RF at 400 mg/kg body weight when co-exposed to magnesium sulphate was highest relative to other treatment groups and loperamide group (Fig. 5).

**Table 4:** Effects of Q3G-RF from *S. mombin* leaves on some parameters of diarrhoea in magnesium sulphate model of Wistar rats

	**Non-diarrhoeal**	**Diarrhoel rats (1 mL of magnesium sulphate)**				
	**Normal saline**	**Normal saline**	**Loperamide**	**Q3G-RF from ** ***S. mombin*** ** leaves (mg/kg body weight)**		
	**1 mL**	**1 mL**	**3 mg/kg body weight**	**100**	**200**	**400**
Onset time (minutes)	50.67 ± 1.04	55.98 ± 1.04^b^	120.55 ± 1.15#	74.65 ± 1.07#	120.59 ± 1.19#	> longest time the experiment lasted
Total number of faeces	1.00 ± 1.01	6.60 ± 1.02^b^	2.00± 0.05#	3.00 ± 0.07#	2.60 ± 0.06#	1.90 ± 0.01#
Number of wet faeces	0.10 ± 0.02	4.40 ± 0.03^b^	0.60 ± 0.03#	1.50 ± 0.02#	0.60 ± 0.04#	-
Fresh weight of faeces (g)	0.15 ± 0.10	3.98 ± 0.07^b^	0.55 ± 0.01#	0.90 ± 0.02#	0.67 ± 0.95#	-
Water content of aces (mL	0.01 ± 0.00	1.59 ± 0.01^b^	0.33 ± 0.04#	0.99 ± 0.08#	0.47 ± 0.11#	-
Inhibition of defecation (%)	-	0	85	64	86	100

Values are mean of 6 replicates ± SEM, significant differences from the sham control group are indicated by ^b^ and from negative control by #, *p* < 0.05

#### Effect of Q3G-RF from S. mombin leaves in rats induced to enteropathy with castor oil

**Fig. 6:**
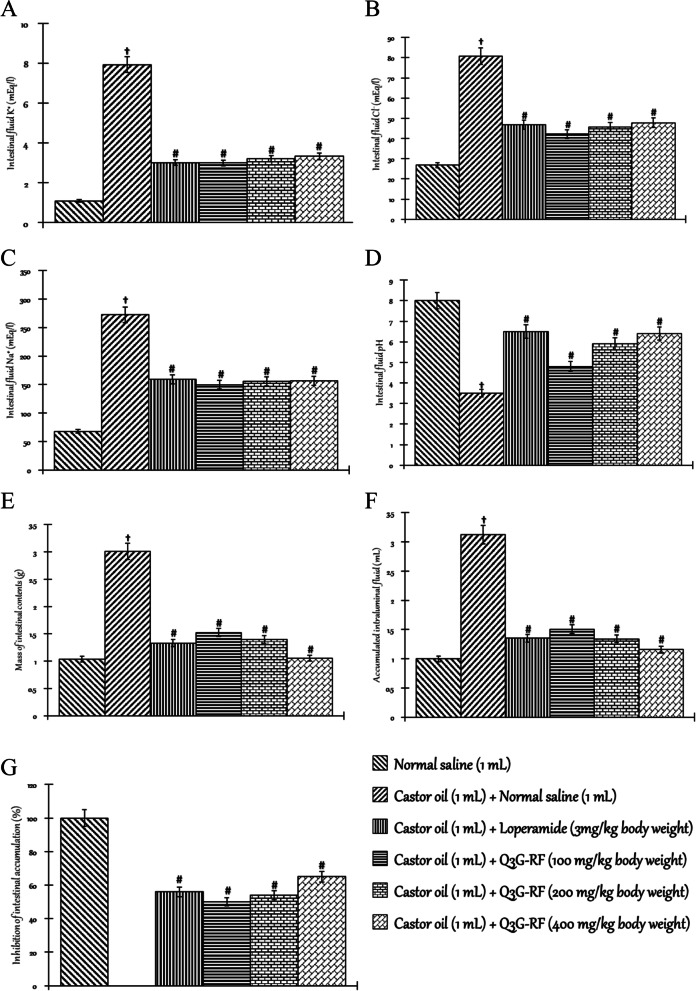
Effects of Q3G-RF from *S. mombin* leaves in castor oil-induced enteropathy rats after 2 h of treatment. Mass of intestinal contents (**A**), accumulated intraluminal fluid (**B**), inhibition of intestinal accumulation (**C**), intestinal fluid Na^+^ (**D**), intestinal fluid K^+^ (**E)**, intestinal fluid Cl^−^ (**F**), and intestinal fluid pH (**G**) of male Wistar rats treated with normal saline, castor oil (1 mL) + normal saline (1 mL), castor oil (1 mL) + loperamide (3 mg/kg body weight), castor oil (1 mL) + Q3G-RF (100 mg/kg body weight), castor oil (1 mL) + Q3G-RF (200 mg/kg body weight), and castor oil (1 mL) + Q3G-RF (400 mg/kg body weight). Values are expressed as mean ± standard error of mean (*n* = 6). Significant differences from the sham control group are indicated by † and from negative control by #, *p* < 0.05

Figure 6 represents the effect of *S. mombin* leaves’ Q3G-RF in rats induced into enteropathy by castor oil. When compared to the non-diarrhoeal animals treated with only normal saline, the mass of intestinal contents (Fig. 6A), accumulated intraluminal fluid (Fig. 6B), and the intestinal fluid Na^+^ (Fig. 6D), K^+^ (Fig. 6E), and Cl^−^ (Fig. 6F) were increased by castor oil (*p* < 0.05). The pH of the intestinal fluid (Fig. 6G) was however reduced by castor oil (*p* < 0.05). However, co-treatment with castor oil and Q3G-RF (100, 200, and 400 mg/kg body weight) or castor oil and loperamide (3 mg/kg body weight) reduced the mass of intestinal contents (Fig. 6A) and accumulated intraluminal fluid (Fig. 6B) as well as the intestinal fluid Na^+^ (Fig. 6D), K^+^ (Fig. 6E), and Cl^−^ (Fig. 6F) (*p* < 0.05), while the pH of the intestinal fluid (Fig. 6G) was increased relative to the diarrhoeal animals treated with only normal saline (*p* < 0.05). In addition, co-treatment with castor oil and Q3G-RF (100, 200, and 400 mg/kg body weight) or castor oil and loperamide (3 mg/kg body weight) resulted in a significant inhibitions of intestinal fluid accumulation (Fig. 6C) when compared with the diarrhoeal animals treated with only normal saline (*p* < 0.05). Co-treatment with castor oil and Q3G-RF at 100 mg/kg, 200 mg/kg, and 400 mg/kg body inhibited the accumulation of intestinal fluid by accumulation of 50%, 54%, and 65%, respectively, while co-treatment with castor oil and loperamide (3 mg/kg body weight) gave 56% inhibitions when compared with the diarrhoeal animals treated with only normal saline (*p* < 0.05). The efficacy of Q3G-RF was most significant with the 400 mg/kg body weight relative to the loperamide-treated animals.

### Effect of Q3G-RF from S. mombin leaves in magnesium sulphate-induced enteropathy rats

**Fig. 7:**
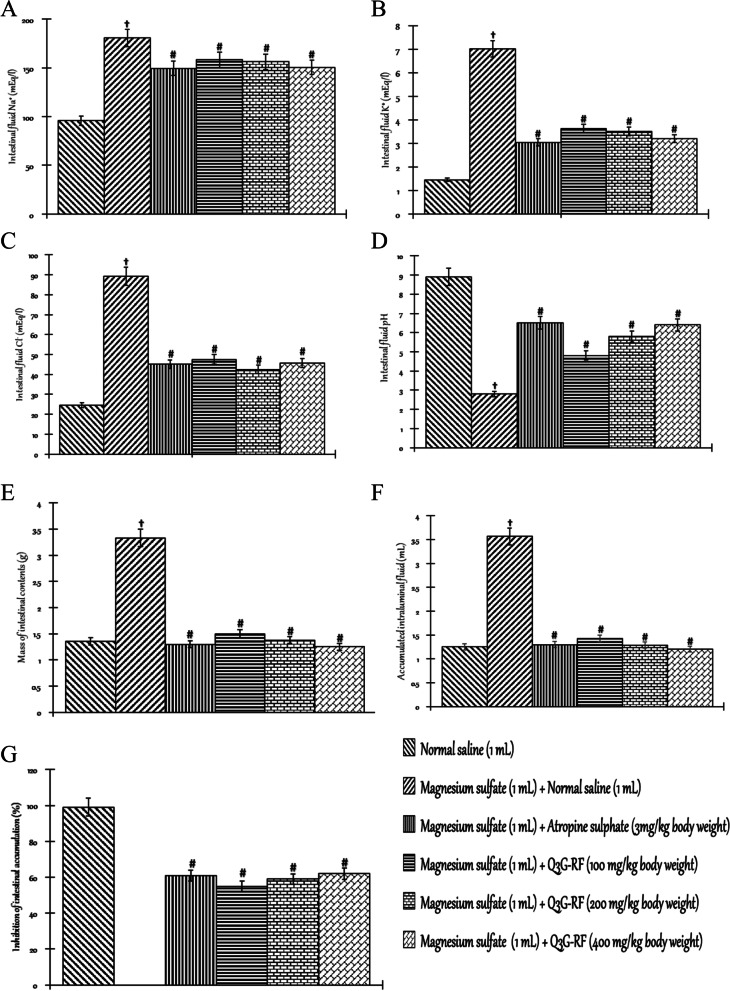
Effects of Q3G-RF from *S. mombin* leaves in magnesium sulphate-induced enteropathy rats after 2 h of treatment. Mass of intestinal contents (**A**), accumulated intraluminal fluid (**B**), inhibition of intestinal accumulation (**C**), intestinal fluid Na^+^ (**D**), intestinal fluid K^+^ (**E**), intestinal fluid Cl^−^ (**F)**, and intestinal fluid pH (**G**) of male Wistar rats treated with normal saline, magnesium sulphate (1 mL) + normal saline (1 mL), magnesium sulphate (1 mL) + atropine sulphate (3 mg/kg body weight), magnesium sulphate (1 mL) + Q3G-RF (100 mg/kg body weight), magnesium sulphate (1 mL) + Q3G-RF (200 mg/kg body weight), and magnesium sulphate (1 mL) + Q3G-RF (400 mg/kg body weight). Values are expressed as mean ± standard error of mean (*n* = 6). Significant differences from the sham control group are indicated by † and from negative control by #, *p* < 0.05

The effect of Q3G-RF from *S. mombin* leaves in rats induced into enteropathy by magnesium sulphate is presented in Fig. 7. The mass of intestinal contents (Fig. 7A), accumulated intraluminal fluid (Fig. 7B), and the intestinal fluid Na^+^ (Fig. 7D), K^+^ (Fig. 7E), and Cl^−^ (Fig. 7F) were significantly (*p* < 0.05) increased by magnesium sulphate relative to the non-diarrhoeal animals treated with only normal saline. The pH of the intestinal fluid (Fig. 7G) was however reduced by magnesium sulphate (*p* < 0.05). Contrastingly, co-treatment with magnesium sulphate and Q3G-RF (100, 200, and 400 mg/kg body weight) or magnesium sulphate and loperamide (3 mg/kg body weight) reduced the mass of intestinal contents (Fig. 7A), accumulated intraluminal fluid (Fig. 7B), and the intestinal fluid Na^+^ (Fig. 7D), K^+^ (Fig. 7E), and Cl^−^ (Fig. 7F) (*p* < 0.05), while the pH of the intestinal fluid (Fig. 7G) was increased, relative to the diarrhoeal animals treated with only normal saline (*p* < 0.05). Furthermore, co-treatment with magnesium sulphate and Q3G-RF (100, 200, and 400 mg/kg body weight) or magnesium sulphate and loperamide (3 mg/kg body weight) resulted in a significant inhibitions of intestinal fluid accumulation (Fig. 7C) when compared with the diarrhoeal animals treated with only normal saline (*p* < 0.05). Co-treatment with magnesium sulphate and Q3G-RF at 100 mg/kg, 200 mg/kg, and 400 mg/kg body weight resulted to a 55%, 59%, and 62% inhibitions of intestinal fluid accumulation respectively, while co-treatment with magnesium sulphate and loperamide (3 mg/kg body weight) gave 61% inhibitions when compared with the diarrhoeal animals treated with only normal saline (*p* < 0.05). The most profound efficacy of Q3G-RF was the 400 mg/kg body weight when compared with the loperamide group.

#### Effect of Q3G-RF from S. mombin leaves on gastrointestinal motility using charcoal

**Fig. 8:**
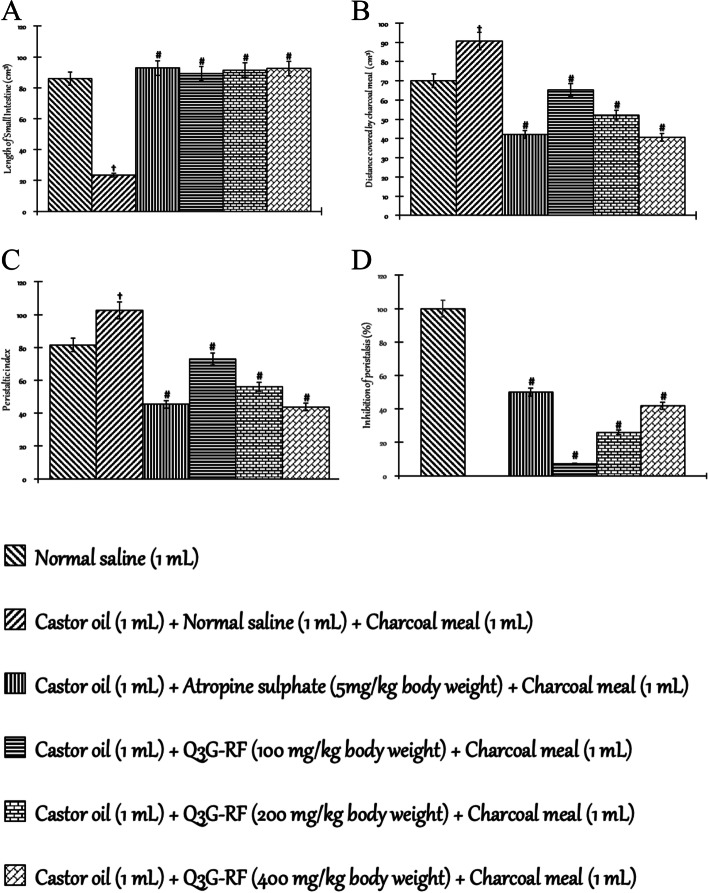
Effect of Q3G-RF from *S. mombin* leaves on gastrointestinal motility using charcoal. Length of small intestine (**A**), distance covered by charcoal meal (**B**), peristaltic index (**C**), and inhibition of peristalsis (**D**) of male Wistar rats treated with normal saline, castor oil (1 mL) + normal saline (1 mL) + charcoal meal (1 mL), castor oil (1 mL) + atropine sulphate (5 mg/kg body weight) + charcoal meal (1 mL), castor oil (1 mL) + Q3G-RF (100 mg/kg body weight) + charcoal meal (1 mL), castor oil (1 mL) + Q3G-RF (200 mg/kg body weight) + charcoal meal (1 mL), and castor oil (1 mL) + Q3G-RF (400 mg/kg body weight) + charcoal meal (1 mL). Values are expressed as mean ± standard error of mean (*n* = 6). Significant differences from the sham control group are indicated by † and from negative control by #, *p* < 0.05

Figure 8 shows the effects of Q3G-RF from *S. mombin* leaves on gastrointestinal motility. Castor oil reduced the small intestinal length (Fig. 8A) and percentage inhibition of peristalsis (Fig. 8D), but the distance covered by the charcoal meal (Fig. 8B) and peristaltic index (Fig. 8C) was increased when compared with the animals treated with only normal saline (*p* < 0.05). In contrast, in animals co-treated with castor oil and Q3G-RF (100, 200, and 400 mgkg^-1^ body) weight), lengths of the small intestine (Fig. 8A) and percentage inhibition of peristalsis (Fig. 8D) were significantly (*p* < 0.05) increased, while the distance travelled by the charcoal meal (Fig. 8B) and peristaltic index (Fig. 8D) was significantly (*p* < 0.05) decreased relative to animals administered with only normal saline. In the same manner as Q3G-RF, the small intestinal length (Fig. 8A) and percentage inhibition of peristalsis (Fig. 8D) were increased, while the distance covered by the charcoal meal (Fig. 8B) and peristaltic index (Fig. 8C) was decreased in animals co-treated with castor oil and loperamide (3 mg/kg body weight), when compared with animals treated with only normal saline (*p* < 0.05).

## Discussion

Due to the increased death rate caused by diarrhoea, researchers are constantly on the lookout for herbal antidiarrhoeal principles and compounds that can reduce the bowel’s transit by slowing propulsion, peristalsis, and segmental contraction. While there are speculations on the folkloric usage of *S. mombin* leaves as diarrhoeal remedy, quercetin-3-O-β-D-glucopyranoside characterized from the leaf extract following chromatographic separation process holds a promising source of compound having prophylactic and therapeutic advantages with multiple targets [23]. Information on the pharmacological activity of *Spondias mombin* leaf fraction rich in quercetin-3-O-β-D-glucopyranoside will be helpful to develop drugs with multiple therapeutic targets against diarrhoea due to infections or other causes like inflammatory bowel disease and functional bowel disorder.

In the present study, three models (infectious, secretory, and osmotic) diarrhoeal was used to evaluate the pharmacological mechanism of Q3G-RF from *Spondias mombin* leaves as an antidiarrhoeal agent. While *Shigella flexneri* secretes verotoxins to facilitate the colonization of the mucus-secreting membrane lining of the colon and easy entry to the closely packed cells forming the epithelium by irritation and decadency of the lamina propria as well as increased rate of cAMP in the intestine epithelium [45], castor oil decompose by reacting with water in the small intestine to ricinoleic acid (its active compound). Ricinoleic acid, via inflammation and injury to the intestinal mucosa, discharges prostaglandin, which in turn induces changes in the transport of electrolytes and the pervasion of the mucosa to elicit secretory and motility diarrhoea. This is done by increasing fluid secretion, decreasing water and electrolyte absorption, decreasing active Na^+^ and K^+^ reabsorption, and decreasing Na^+^–K^+^ ATPase [46, 47]. Increased peristaltic activity alteration in the transport of electrolytes and water is among the side effects of castor oil, which also causes constriction of intestinal smooth muscles and an increase in intestinal content volume by blocking water reabsorption [46]. Therefore, ricinoleic acid causes a chain reaction that ultimately leads to diarrhoea in humans by modulating the gastrointestinal tract, stimulating motility and secretion, and causing diarrhoea [38]. This chain reaction involves the release of eicosanoids, platelet-activating factor, cyclic AMP, histamine, nitric oxide, tachykinins, and, most importantly, endogenous prostaglandins [48]. Magnesium sulphate has also been reported to increase intestinal content volume via prevention of reabsorption of water, therefore inducing diarrhoea. Natural compounds, such as Q3G-RF from *Spondias mombin* leaves, can therefore be investigated as potential treatments for diarrhoea owing to their efficacy to inhibit or slow down the biosynthesis of infectious, secretory, and osmotic diarrhoea.

Increase in electrolytes and intestinal fluid secretion and the reduced absorbed fluid, electrolytes, and nutrients (in few times in the small and large intestine) are the two major ways that perturb the normal intestinal physiology because of infectious diarrhoea. In this study, increasement in intestinal fluid secretion as well as concentration of Na^+^, K^+^, Cl^−^, glucose, and total protein denotes infectious diarrhoea by *Shigella flexneri*, while the increase in Na^+^, K^+^, and Cl^−^, as well as glucose and total protein by Q3G-RF, lays credence to the efficacy in absorption of fluid and electrolytes in the small intestine. Q3G-RF from *S. mombin* leaves might have possibly demonstrated its pharmacological activity against *Shigella flexneri* effect by reversing the ulceration of the mucosa and further enhancing reabsorption in the intestinal lumen to prevent elsewise loss of mucus and blood in the intestinal lumen. Q3G-RF being a polyphenolic compound might have acted to stimulate the macrophages and dendritic cells to produce active metabolites or agents to phagocytically digest the bacteria. In this regard, Q3G-RF can be said to demonstrate immune-stimulating properties that can bind the toll-like receptors to enhance the immune system against infectious bacteria like *Shigella flexneri* [49]. The significant weight changes in the rats co-administered *Shigella flexneri* inoculum, and Q3G-RF connotes its capability to influence growth, immune functionality, and decreased susceptibility to infections.

The reduction in faecal parameters (faecal water content and fresh weight, stool frequency, wet feacel number, and total feacal number) as well an increase in less frequent and less watery faeces, in this study, potentiates the antidiarrhoeal effect of Q3G-RF from *S. mombin* leaves owing to its ability to inhibit the production of prostaglandin [50, 51]. Compounds in Q3G-RF might have relaxed the smooth muscle, constricted the nerves and muscles, or inhibited the mucosity of the small intestine. Moreover, suppression of nitric acid in this study also corroborates the prostaglandin inhibitory activity by Q3G-RF, as nitric acid elicits anti-inflammatory capacity on alimentary canal and prevents water and electrolytes reabsorption [50, 52]. Suppression of nitric oxide is one of the probable modes of action by which Q3G-RF exhibits its antidiarrhoeal properties as anti-inflammation compound. The significant reduction in the concentrations of sodium ion, potassium ion, chloride ion, and glucose indicates that the mucus-secreting membrane lining of the intestine was protected from being irritated and inflamed by castor oil. The possibility of this can be attributed to Q3G-RF’s ability to inhibit the synthesis/release of prostaglandins or production of platelet-activating factors for steady water and electrolytes discharge [52], spontaneous movement, or easy transfer of water and electrolytes [53] by direct influence on the colonic absorptive process. Furthermore, the ability of Q3G-RF from *S. mombin* leaves to increase the activity of Na^+^–K^+^ ATPase in the intestinal tissue contrary to ricinoleic acid action (the active agent in castor oil that hinders a spontaneous electrolytes transfer and easy permeation of sodium and potassium ions, consequentially limiting the rate of Na^+^–K^+^ ATPase) additionally substantiate its antidiarrhoeal activity [54]. Q3G-RF might have enhanced the Na^+^–K^+^ ATPase action via the de novo synthesis or possibly by influencing the NO/prostaglandin pathway [55], which explains why Na^+^–K^+^ ATPase rate was more than that of loperamide, a common and highly effective antidiarrhoeal medication that blocks or slows down intestinal [48]. This suggests that Q3G-RF could act against magnesium sulphate mechanism to reduce intestinal secretion (couple with reduction of cholecystokinin from the duodenal mucosa) and motility that promotes increased reabsorption of sodium, chloride, and water [56].

Antienteropooling activity of Q3G-RF from *S. mombin* leaves was demonstrated by its ability to decrease both intestinal content mass and volume in castor oil and magnesium sulphate-induced enteropathy. By inhibiting the secretion of electrolytes and water in the small intestine, Q3G-RF prevented the accumulation of induced intestinal fluid. These pharmacological effects suggest that Q3G-RF may improve water and electrolytes reabsorption from intestinal lumen [48]. This may demonstrate improved lysis and assimilation of water and electrolytes, corroborated by the fact that Q3G-RF increased Na^+^–K^+^ ATPase rate in this study [38].

Furthermore, ability of Q3G-RF from *S. mombin* leaves demonstrated by repressing the propellant bowel movement/euphemism for defecation or GI movement of charcoal meal connotes its stool frequency-lowering capability. This by extension supports the lengthening time required for water and electrolytes permeation in diarrhoeal conditions. In the present study, decrease in peristaltic activity may have contributed to decreased gastrointestinal motility and the computed peristaltic index. Since substances that block the action of acetylcholine at a receptor site also inhibit excessive motility of the gastrointestinal tract [38], Q3G-RF from *S. mombin* leaves can be said to inhibit gastrointestinal disorders in the diarrhoeal condition through an anticholinergic effect [23].

The antidiarrhoeal activity of Q3G-RF in the present study may be credited to its phytoconstituents (secondary metabolites) in *Spondias mombin* leaves particularly flavonoids. Flavonoids and saponins prevent contractions, motility, and hydroelectric secretions and hinder the release of spasmogenic autocoids and prostaglandins [57], whereas tannins and polyphenols reduce intestinal transit and secretion, encourage intestinal mucosal resistance, and help to normalize disrupted water transport across the mucosal cells [58]. Antidiarrhoeal terpenoids and alkaloids work by slowing or stopping peristaltic movement of the intestine, allowing more time for the body to absorb water and electrolytes. In particular, alkaloids have a suppressive effect on GI motility [59]. Because of their ability to increase membrane permeability, saponins has the ability to inhibit the release of histamine [60]. The pharmacological properties of *Spondias mombin* leaves may be increased by enteric synthesis and release of fluid and electrolytes or decreased permeation of fluid and electrolytes via any of these aforementioned mechanisms (Verma et al., [48]; Yakubu and Salimon, [38]). In this research, the antidiarrhoeal efficacy of *Spondias mombin* leaves can be traced to the flavonoid constituents in the quercetin-3-O-β-D-glucopyranoside-rich fraction [61]. These gives a scientific ground for the possible exploration of quercetin-3-O-β-D-glucopyranoside (Q3G) as curative agent against infectious, secretory, and osmotic diarrhoeal conditions.

## Conclusions

**Scheme 1:**
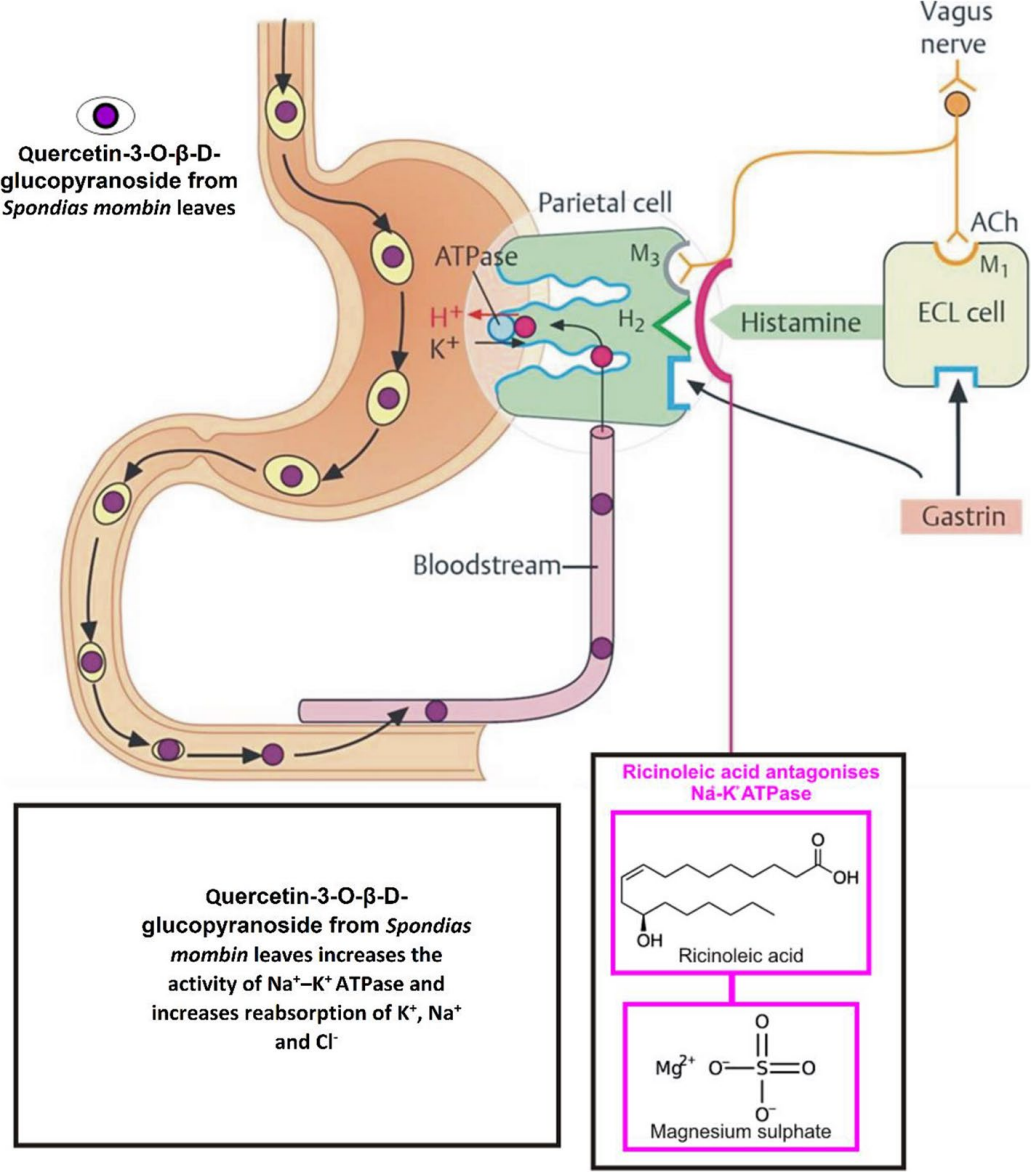
Quercetin-3-O-β-D-glucopyranoside stimulates Na^+^–K^+^ ATPase, inhibits prostaglandins, or suppresses nitric oxide as antidiarrhoeal agent

Overall, findings from this study supported the pharmacological activity of quercetin-3-O-β-D-glucopyranoside from *Spondias mombin* leaves against infectious, secretory, and osmotic form of diarrhoeal and further justified its traditional use in the treatment of diarrhoea due to its antimotility, antisecretory, and antimicrobial properties (Scheme 1). Because of its polyphenolic properties, quercetin-3-O-β-D-glucopyranoside can stimulate Na^+^–K^+^ ATPase, inhibit prostaglandins, or suppress nitric oxide. This means that quercetin-3-O-β-D-glucopyranoside from *Spondias mombin* can be investigated as a potential antimotility, antisecretory, and antimicrobial agent for the treatment of functional bowel disorder, inflammatory bowel disease, or infectious diarrhoea by gastroenterologist and policy makers.

## Data Availability

The raw data can be tendered upon reasonable request.

## Abbreviations

Q3G-RF: Quercetin-3-O-β-D-glucopyranoside
WHO: World Health Organization
OECD: Economic Co-operation and Development
LD_50_: Lethal dose
CFU: Colony-forming units
